# Supplementary material for: Gut Microbiome Changes in Patients with Active Left-Sided Ulcerative Colitis after Fecal Microbiome Transplantation and Topical 5-aminosalicylic Acid Therapy
Source: Cells. 2020 Oct 13;9(10):2283. doi: 10.3390/cells9102283 (PMC7602113; doi:10.3390/cells9102283)
Supplement: Supplementary file 1 [file cells-09-02283-s001.pdf]

Supplementary Material

Gut microbiome changes in patients with active left-sided ulcerative colitis after fecal microbiome transplantation and topical 5-aminosalicylic acid therapy

Dagmar Schierová [1*], Jan Březina [2], Jakub Mrázek [1*], Kateřina Olša Fliegerová [1], Simona Kvasnová [1], Lukáš Bajer [2] and Pavel Drastich [2]

[1] Institute of Animal Physiology and Genetics of the Czech Academy of Science, v.v.i., Prague, Czech Republic

[2] Hepatogastroenterology Department, Institute for Clinical and Experimental Medicine, Prague, Czech Republic

* Correspondence: MJ mrazek@iapg.cas.cz, SD schierova@iapg.cas.cz

Table S1: Patient inclusion and exclusion criteria

**Patient inclusion criteria**	**Patient exclusion criteria**
Left-sided ulcerative colitis	Anti-TNF medication in the previous 6 months
UC affecting more than 15 cm	Cyclosporine in the previous 4 weeks
UC ongoing more than 3 months	Methotrexate in the previous 2 months
Age ≥18	Prednisone > 10 mg
Mayo score < 10	CMV infection
Endoscopic Mayo score ≥ 2	Positive stool culture (Salmonella, Shigella, Yersinia, Campylobacter, pathogenic *E. coli*, *Clostridium difficile*)
	Pregnancy and breastfeeding

Table S2: Donor inclusion and exclusion criteria

Donor inclusion criteria	Donor exclusion criteria
**Negative blood test**: hepatitis A, B, C, HIV-1, HIV-2, Treponema pallidum,	Treatment with antibiotics or PPI in the last 6 months
**Negative blood test for active infection**: cytomegalovirus, Epstein-Barr, herpes simplex,	History of chronic gastrointestinal problems (IBD, constipation, functional dyspepsia)
Varicella zoster	History of autoimmune disease
**Negative stool tests**: Yersinia spp., Salmonella spp., Shigella spp., *Campylobacter jejuni*, *C. difficile* toxin, *Helicobacter pylori*	Immunosuppression
Standard parasitological examination	Hospitalization in the last 3 months
	Obesity

Table S3: Stool sample collection scheme

Patient	Therapy	Responder	Gender	Baseline	Week 2	Week 4	Week 6	Week 12
P02	FMT	non-responder	F	●	●	●	●	●
P04	FMT	responder	M	●	●	●	●	●
P08	FMT	responder	M	●	●	●	●	●
P09	FMT	non-responder	F	●	●●	●	●	●●
P11	FMT	non-responder	F	●	●	●	●	●
P14	FMT	non-responder	M	●	●	●	*	*
P16	FMT	non-responder	M	●	●	●	●	*
P21	FMT	responder	F	●	●	●	●	●
P01	5-ASA	non-responder	M	●	●			●
P03	5-ASA	responder	M	●	●			●
P06	5-ASA	responder	F	●	●			●
P07	5-ASA	non-responder	F	●	●			*
P12	5-ASA	non-responder	M	●	●			●
P17	5-ASA	non-responder	F	●	●			*
P18	5-ASA	responder	F	●	●			●
P19	5-ASA	responder	M	●	●			*

* High content of blood and fluids or lack of the sample caused DNA extraction impossible, no 16S data acquired.

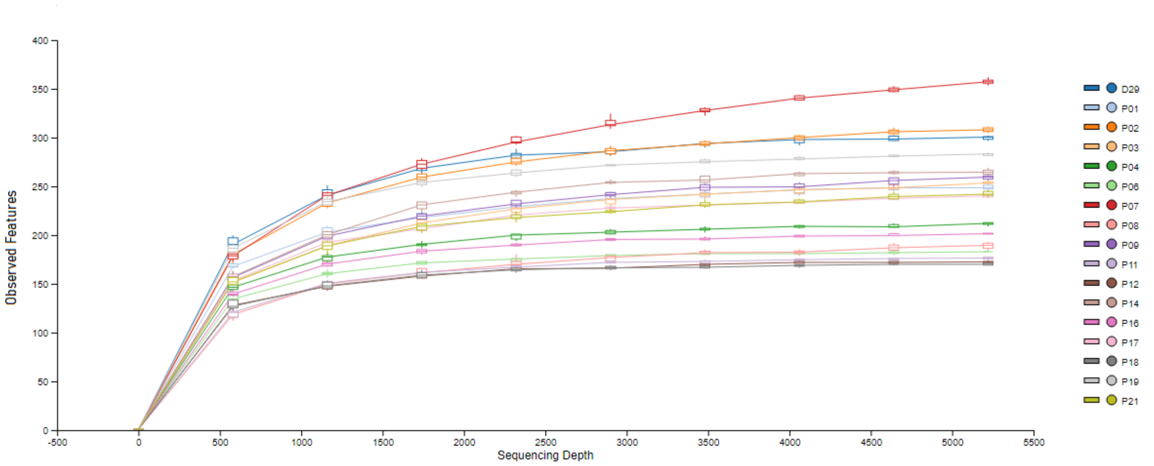

Figure S1: Rarefaction curves showing sequencing depth (number of reads) and the average number of features (sequence variants) found in the stool samples of patients with active left-sided UC (*n*=16) and a donor (*n*=1). Patients are indicated by the letter P, donor by letter D.

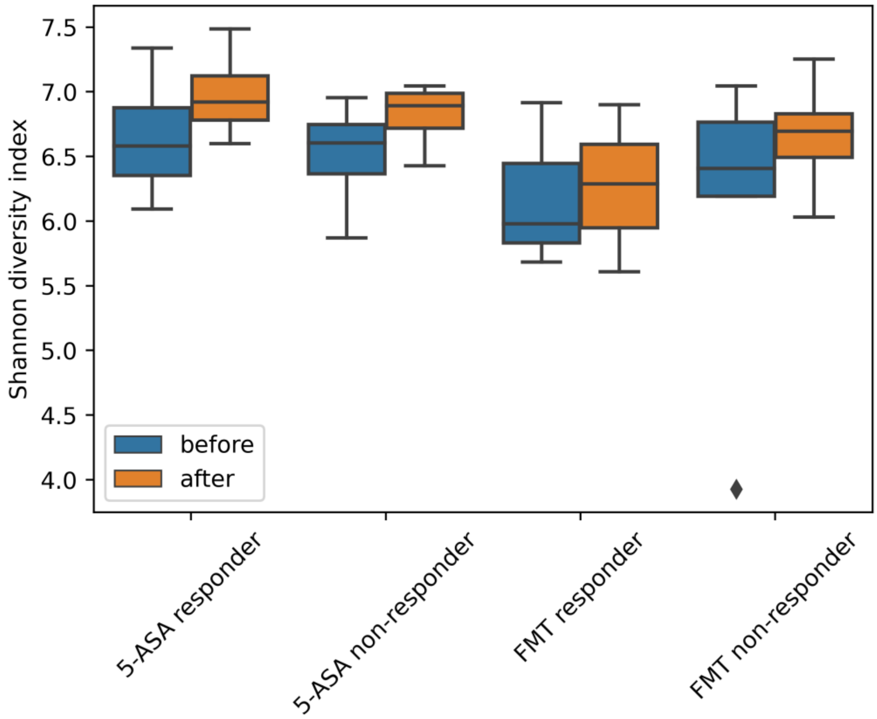

Figure S2: Alpha diversity represented by Shannon diversity index in the patients with active left-sided ulcerative colitis for FMT (*n*=8) and 5-ASA (*n*=8) groups showing responders and non-responders before the treatment and at all sampling points after the start of treatment.

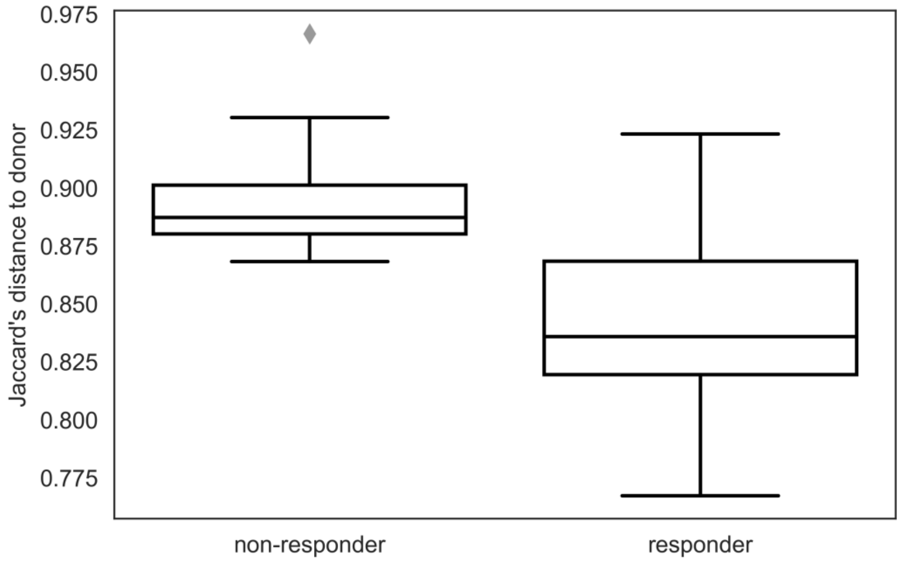

Figure S3: Box plots showing Jaccard's distance of patients with active left-sided ulcerative colitis to donor, which was significantly different between FMT responders and FMT non-responders (p = 0.00003). Analysis, including all sample points after the start of the FMT therapy, shows that non-responders were more distant from the donor, indicating that their fecal microbiomes were less similar to the donor's microbiome than to the microbiome of responders.

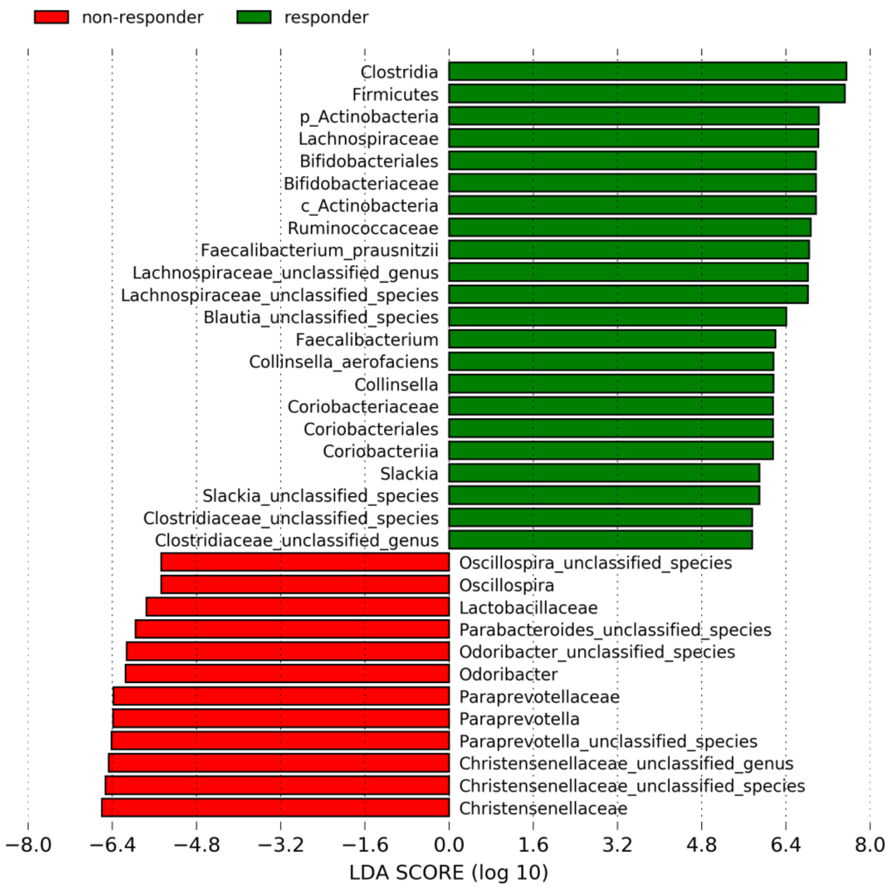

Figure S4: Linear discriminant analysis scores of responders and non-responders in the FMT group of patients with active left-sided ulcerative colitis on different taxonomical levels (phylum, class, order, family, genus, and species) for all sampling points including baseline. To distinguish Actinobacteria phylum and class, we used shortcuts p and c, respectively.

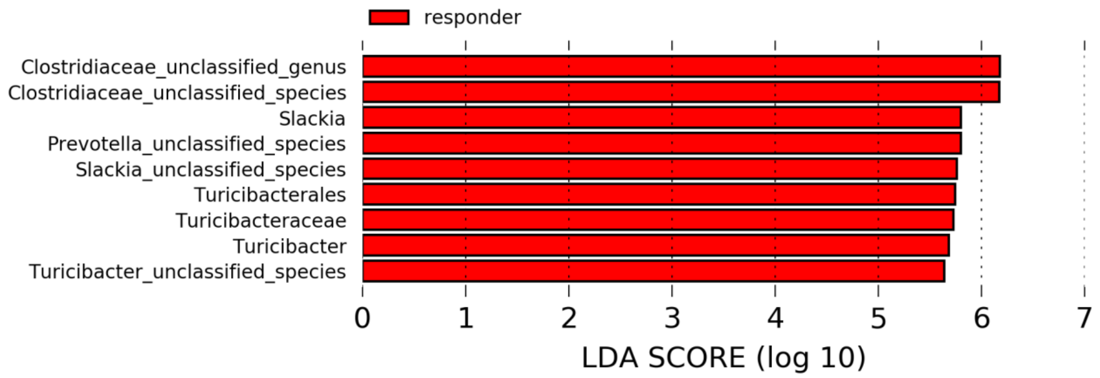

Figure S5: Linear discriminant analysis scores of responders in the FMT group of patients with active left-sided ulcerative colitis on different taxonomical levels (order, family, genus, and species) for week 2 after treatment initiation. Non-responders did not show LDA score > 2, and hence are not shown.

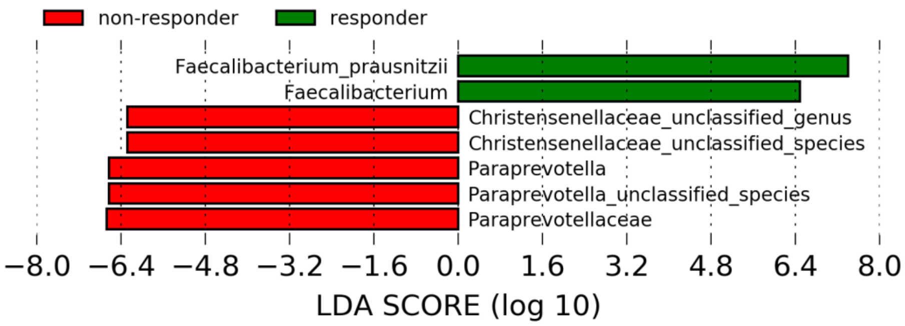

Figure S6: Linear discriminant analysis scores of responders and non-responders in the FMT group of patients with active left-sided ulcerative colitis on different taxonomical levels (family, genus, and species) for week 4 after treatment initiation.
